# Redox Balance in Type 2 Diabetes: Therapeutic Potential and the Challenge of Antioxidant-Based Therapy

**DOI:** 10.3390/antiox12050994

**Published:** 2023-04-25

**Authors:** Lital Argaev-Frenkel, Tovit Rosenzweig

**Affiliations:** 1 Department of Molecular Biology, Ariel University, Ariel 4070000, Israel; litalfren@gmail.com; 2 Adison School of Medicine, Ariel University, Ariel 4070000, Israel

**Keywords:** oxidative stress, reductive stress, redox balance, antioxidant, type 2 diabetes

## Abstract

Oxidative stress is an important factor in the development of type 2 diabetes (T2D) and associated complications. Unfortunately, most clinical studies have failed to provide sufficient evidence regarding the benefits of antioxidants (AOXs) in treating this disease. Based on the known complexity of reactive oxygen species (ROS) functions in both the physiology and pathophysiology of glucose homeostasis, it is suggested that inappropriate dosing leads to the failure of AOXs in T2D treatment. To support this hypothesis, the role of oxidative stress in the pathophysiology of T2D is described, together with a summary of the evidence for the failure of AOXs in the management of diabetes. A comparison of preclinical and clinical studies indicates that suboptimal dosing of AOXs might explain the lack of benefits of AOXs. Conversely, the possibility that glycemic control might be adversely affected by excess AOXs is also considered, based on the role of ROS in insulin signaling. We suggest that AOX therapy should be given in a personalized manner according to the need, which is the presence and severity of oxidative stress. With the development of gold-standard biomarkers for oxidative stress, optimization of AOX therapy may be achieved to maximize the therapeutic potential of these agents.

## 1. Introduction

Oxidative stress is an imbalance between the generation and neutralization of Reactive Oxygen Species (ROS) in favor of the accumulation of oxidants. ROS are defined as oxygen-containing reactive species. Among this family of molecules or molecular fragments, some are highly reactive free radicals, containing one or more unpaired electrons, which increase their reactivity with other molecules. Several other ROS, including H_2_O_2_, do not contain unpaired electrons and thus are non-radical oxidizing agents [1]. The varied ROS differ vastly in their molecular targets and levels of reactivity [2,3] and may interact with each other to produce other reactive molecules or react with a variety of proteins, nucleic acids and lipids, altering their structure and function while forming new reactive species to generate a chain reaction [3,4]. Most intracellular ROS are naturally generated as byproducts of the mitochondrial respiratory chain. An incomplete electron transfer, mainly through electron complexes I and III, reduces O_2_ to form superoxide, which might be converted to H_2_O_2_ by the activity of superoxide dismutase (SOD) [5]. ROS are also generated as the main product of several physiological processes, such as the enzyme activity of the NADPH oxidase (Nox) family [1].

The free-radical theory of aging, proposed by D. Harman [6], suggests that aging and various age-related chronic diseases, such as atherosclerosis, neurodegenerative diseases and Diabetes mellitus Type 2 (T2D), are induced or intensified by the cumulative damage induced by ROS. With the recognition of the harm induced by ROS, it was proposed that the use of antioxidants to prevent or to slow their production might impair the development of these age-related diseases. Actually, the commercial market of antioxidant (AOX) supplements has flourished, currently being valued at over 3 billion USD a year (by 2020), and is still on the rise [7]. Unfortunately, the free-radical theory of aging was challenged by disappointing results, showing that AOXs fail to prevent the prevalence of certain oxidative stress-associated diseases or to attenuate their severity [8,9,10]. Moreover, some findings were reported that raise concerns regarding the safety of AOX supplementation in certain situations [11]. These observations led to the recognition of the important role of ROS in several physiological functions. Accordingly, the free-radical theory of aging had been rephrased accurately as the ‘oxidative-stress theory of aging’, emphasizing the idea that ROS are damaging agents only upon their pathological accumulation. Accordingly, it was concluded that the degree of redox balance can range from physiological oxidative stress (eustress), which is a temporal event, to an excessive and toxic oxidative burden (distress) [12,13]. Thus, in line with the principle coined by Paracelsus—‘The dose makes the poison’—ROS are involved in both physiological and pathological processes, in dependency on the ability to maintain a delicate redox balance.

Type 2 diabetes (T2D) is a ubiquitous metabolic disorder, affecting more than 500 million adults according to numbers available for 2021 [14]. The disease is characterized by inadequate glucose homeostasis and is intimately linked to obesity, which is considered its major risk factor. T2D is developed from a combination of several pathophysiological factors, including insulin resistance and pancreatic β-cell dysfunction, as well as metabolic inflammation and oxidative stress. The interaction between oxidative stress and T2D is bi-directional; oxidative stress may result in a response to metabolic alterations associated with T2D, but has also been implicated as a contributor to both onset and progression of T2D and its associated complications [15]. Unfortunately, a summary of several decades of research indicates that AOX therapy on glucose homeostasis in diabetic subjects may be disappointing at best. The complexity of ROS effects, in both the physiology and pathophysiology of glucose homeostasis, appears to complicate the potential benefits of AOX therapy and might explain the disappointing results of AOXs in the prevention and treatment of T2D [16].

In this review, we will discuss the bidirectional effects of ROS in the various systems that are involved in regulation of glucose balance. We will briefly summarize the involvement of ROS in the pathophysiology of T2D, the evidence for the effects of AOXs in the prevention and treatment of diabetes, and will discuss possible explanations for the failure of AOX therapy in T2D.

## 2. The Role of Oxidative Stress in the Pathophysiology of Type 2 Diabetes

### 2.1. Oxidative Stress Is Developed in T2D

An extensive literature has demonstrated the presence of oxidative stress in diabetic patients. Increased levels of molecules generated by oxidation reactions, such as products of lipid peroxidation and protein carbonylation, were found in plasma and urine of T2D patients [17,18,19,20,21]. In addition, higher levels of oxidation products were detected in adipose tissue, liver, and muscle of diabetic animals [22,23].

Many of the common risk factors for T2D, such as obesity, inflammation, and an unhealthy diet, contribute to an oxidative environment. Chronic inflammation, detected in most obese subjects and subjects with T2D, is accompanied by ROS generation [24]. In addition, metabolic overload, associated with obesity and T2D and manifested by elevated glucose and free fatty acids (FFA), was found to increase intracellular ROS accumulation in pancreatic β cells and in insulin target tissues [25,26]. The energy overload, and specifically an oversupply of glucose, enhances oxidative respiration, eventually leading to an increase in mitochondrial ROS, which are by-products of the electron transfer chain. Specifically, pancreatic β-cells are highly affected by high glucose flux, since pancreatic islets are perfused with a dense microcirculation that supplies a relatively high blood flow. Moreover, a high expression of Glut2, a transporter that carries glucose according to its concentration gradient, makes these cells highly affected by hyperglycemia and the production of mitochondrial ROS [27].

Another significant source of ROS generation in β-cells is the increasing demand for insulin, as occurs in conditions of insulin resistance, which leads to elevated synthesis of this hormone. This is because insulin processing requires the generation of disulfide bonds, a process that produces large amounts of ROS, depletes the GSH pool [28,29] and eventually generates oxidative stress in pancreatic beta cells. Thus, several different pathophysiological events associated with T2D promote the development of oxidative stress in different related organs.

**Figure 1:**
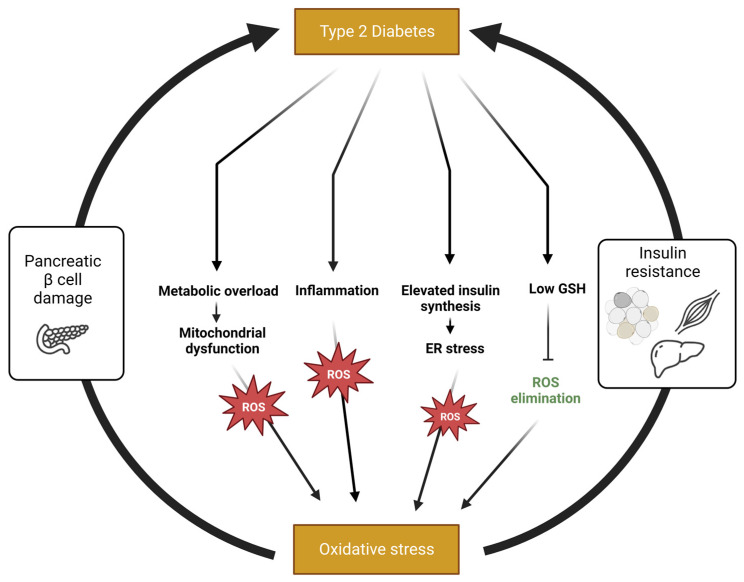
The role of oxidative stress in the pathophysiology of T2D. Oxidative stress is developed in T2D as a result of low GSH and increased ROS generation due to (1) metabolic overload and the consequential mitochondrial dysfunction, (2) inflammation and (3) the increasing demand for insulin synthesis, resulting in ER stress. Oxidative stress, in turn, induces insulin resistance and impairs pancreatic β cell viability and function, leading to an additional worsening of the pathology. Created with BioRender.com (accessed on 5 March 2023).

In addition to elevated ROS generation, diminished activity of the endogenic AOX system might contribute to ROS accumulation and oxidative stress development. However, evidence regarding the expression and activity of AOX enzymes in diabetic patients is inconsistent and depends on the specific AOX enzyme measured, tissue of interest and severity of the disease. Thus, while some studies showed a decrease in the expression of AOX enzymes (including SOD, catalase and Gpx), others reported the opposite [30]. The inconsistency is at least partially attributed to the fact that oxidative stress might increase the activity of AOX systems as an adaptive response [31]. Regarding levels of GSH, the principal intracellular AOX, many studies showed a negative correlation between its levels and blood glucose or the severity of diabetes [31,32]. Depletion of GSH in T2D might result from high demand for this AOX upon oxidative stress, which induces irreversible utilization of GSSG and a lack of both GSH and GSSG [33]. In addition, impaired recycling of GSSG into GSH also contributes to GSH depletion in T2D. Poor recycling results from a deficiency in NADPH, a cofactor of glutathione reductase, due to the high utilization rate of NADPH in the sorbitol/polyol pathway, under hyperglycemic conditions [34]. Moreover, several studies demonstrated that synthesis of GSH is impaired in T2D as a result of either limited substrate availability or dysfunction of enzymes required for GSH synthesis, such as gamma-glutamyl transpeptidase, glutamine-cysteine ligase and glutathione synthetase [33,35,36]. Therefore, oxidative stress is developed in T2D as a result of both elevated ROS generation and insufficient ROS neutralization (Figure 1).

### 2.2. Oxidative Stress Contributes to the Progression of T2D

Oxidative stress is promoted by several pathological factors associated with T2D. Unfortunately, oxidative stress by itself has an additional negative impact on glucose homeostasis and is suggested to be a causal factor of the disease, not merely a consequence of it [37,38]. Some of the negative outcomes of an oxidative environment that promotes the development of T2D are impaired glucose tolerance, impaired insulin signaling, β-cell dysfunction, and mitochondrial dysfunction [39]. Thus, the presence of oxidative stress lead to an additional deterioration in the functionality of the physiological systems required for maintaining glucose balance (Figure 1).

#### 2.2.1. Oxidative Stress as a Cause of Insulin Resistance

Insulin resistance is defined as an impairment of insulin action in its target tissues, including mainly the liver, adipose tissue and muscle. Oxidative stress has been implicated as a major contributor to insulin resistance. Prolonged exposure of various cell types to H_2_O_2_ at the millimolar range inhibits insulin action, decreases insulin-induced glucose transport and membrane translocation of GLUT-4 [39,40,41]. The exact mechanism by which oxidative stress impairs insulin signaling is not fully understood, but several mechanisms have been proposed. Oxidative stress increases IRS1 serine phosphorylation, which disrupts the ability of an IRS protein to interact with an insulin receptor and impairs the protein’s ability to recruit and activate downstream components of the cascade. This leads to suppression of insulin-induced glucose transport [42]. In addition, activation of stress-sensitive Ser/Thr MAPKs, such as c-Jun N-terminal kinase (JNK) and p38 pathways [37,42,43,44], which are negative regulators of insulin signaling, is suggested to mediate the inhibitory effect of oxidative stress on insulin action. Oxidative stress-dependent alterations in gene expression may also affect insulin signaling. Several transcription factors have been found to be regulated by oxidative stress, including NFκB, NF-1 and AP-1 [45,46], leading to reduced GLUT4 expression [45,47].

Another mechanism mediating the adverse effect of ROS on insulin sensitivity is the development of mitochondrial dysfunction [48]. Most intracellular ROS are generated as byproducts of oxidative phosphorylation in mitochondria, with an elevated rate of ROS production under conditions of an imbalance between nutrient supply and ATP requirements. The accumulated ROS damage the mitochondria, leading to deterioration of the mitochondrial capability of fuel oxidation. Reduced numbers and sizes of mitochondria, and an abnormally low activity of the respiratory chain, were observed in target tissues of insulin in T2D subjects [28,49,50,51]. In addition to elevated ROS production, an impaired mitochondrial capacity leads to the accumulation of lipid intermediates, such as ceramides that suppress insulin signaling through inhibition of Akt, and diglycerides, which are allosteric activators of PKC-β, δ, and θ, that phosphorylate IRS on serine residues, leading to inhibition of transmission of the insulin cascade [52].

#### 2.2.2. Oxidative Stress Impairs Insulin Secretion

As mentioned, hyperglycemia and insulin resistance lead to elevated ROS production in β-cells as a result of an increased rate of oxidative phosphorylation and insulin synthesis, respectively [28]. However, while β-cells are exposed to elevated levels of ROS, these cells have a weak antioxidant defense system compared to other tissues [25,53,54,55]. Eventually, the imbalance of ROS production with their dissimilation leads to oxidative stress, impaired β-cell function and reduced β-cell mass [56,57]. Activation of the JNK pathway is a major mechanism mediating the adverse effects of oxidative stress on β-cells. Elevated activity of JNK disturbs nuclear localization of the pancreatic and duodenal homeobox 1 (PDX-1) transcription factor, leading to decreased insulin gene expression, lower insulin content in β-cells, and reduced insulin secretion [57,58]. In addition, JNK activation, along with some additional stress pathways (p38 and p53), induces apoptosis of β-cells in response to oxidative stress [59,60].

## 3. Oxidative Stress Contributes to Hyperglycemia-Induced Diabetic Complications

Beyond its involvement in the pathophysiology of T2D, ROS has been closely implicated in the development of diabetic complications [61]. It is well known that hyperglycemia causes tissue damage, which renders diabetic patients highly susceptible to developing microvascular complications, including retinopathy, nephropathy and neuropathy, and macrovascular phenomena, such as cardiovascular diseases. Oxidative stress mediates at least part of the destructive outcomes of hyperglycemia. Accumulated ROS oxidize various types of molecules, modifying their structure and functionality, thus leading to extensive and non-specific tissue damage. In addition, hyperglycemia-induced oxidative stress activates certain specific harmful mechanisms including the (1) sorbitol/polyol pathway, (2) hexosamine pathway, (3) increased levels of AGEs and their receptor (RAGE), and (4) activation of certain PKC isoforms. All these mechanisms contribute further to the development of diabetic complications and are involved in diabetic vascular damage [61,62]. These deleterious effects of ROS are mediated through oxidative post-translational modifications of the glycolytic enzyme glyceraldehyde-3 phosphate dehydrogenase (GADPH), leading to its inhibition [63]. As a result of GAPDH inhibition, there is an accumulation of all upstream glycolytic intermediates, such as glyceraldehyde-3-phosphate (GA3P) and fructose-6 phosphate (F6P), as well as an increase in glucose levels [64,65]. The elevated level of these intermediates stimulates all four pathways mentioned above; the sorbitol/polyol pathway is stimulated by high glucose levels, the hexosamine pathway is stimulated by elevated F6P, while high GA3P, being a precursor of glyceraldehyde and methylglyoxal, promotes the advanced glycation end-products (AGE) pathway [66]. In addition, GA3P might be converted to glycerol-P, leading to an increased de-novo synthesis of diacylglycerol and activation of classic protein kinase C (PKC) isoforms [67], which are implicated in cardiovascular pathologies [68].

Diabetic vascular complications are also mediated by oxidative stress-induced alterations in endothelial cyclooxygenases (COXs) activity. Enzymes of the COX family produce prostanoids that act as contracting or relaxing factors. Oxidative stress alters the activity of these enzymes, with an upregulation of COX2, favoring the generation of contracting prostanoids and the resulting increase in vascular tone [69].

Another mechanism linking oxidative stress to diabetic complication is the ROS-dependent upregulation of VEGF, which leads to pathological angiogenesis and contributes largely to diabetic retinopathy [70,71]. In addition, elevated ROS stimulate pro-inflammatory pathways, leading to inflammation, which has a role in the development of retinopathy, nephropathy and neuropathy [72,73,74,75].

## 4. AOX for the Management of Diabetes: Limited Benefits in Clinical Studies

Given the contribution of oxidative stress to the onset and progression of diabetes, it would be reasonable to expect that AOX therapy might be beneficial for the prevention of T2D. In addition, it was expected that AOX supplements would improve glycemic control and reduce the risk of complications when given as an add-on therapy to anti-diabetic drugs. Since T2DM leads to severe complications in several distinct organs, it is assumed that AOX treatment will be effective as a whole-body treatment attenuating ROS in parallel in many organs. Accordingly, a large number of studies, utilizing in vitro and in vivo models of diabetes, demonstrated the benefits of AOXs in restoration of glucose homeostasis. For example, augmentation of antioxidant defense in animal models of diabetes, achieved by enhancing antioxidant enzyme activity or by dietary supplementation was shown to improve markers of insulin sensitivity [76,77,78,79,80,81,82,83]. It was also shown that increasing activity of the antioxidant systems in pancreatic β cells, either through NAC administration or overexpression of AOX enzymes, affords protection from ROS and preserves their function and survival [79,84,85,86,87].

Exogenous administration of AOX enzymes or their mimetics had anti-diabetic effects in cellular and rodent models [88]. Liposome-embedded SOD or SOD mimics improved glucose tolerance and insulin sensitivity in diet-induced obese mice and diabetic rats [89,90,91], attenuated the progression of diabetic nephropathy in T2D mice [92], and protected β-cells from oxidative damage [93]. Similarly, ebselen, a GPx mimic, also improved insulin sensitivity [94], preserved pancreatic β-cell function in diabetic rodents [95] and prevented diabetes-related atherosclerosis and renal damage [96].

In contrast, however, to the vast support from in vitro and in vivo experiments for the potential of AOX therapy for the treatment of diabetes, many clinical studies failed to validate such benefits of AOX supplementation [97]. The inconsistency in clinical trials regarding the effectivity of AOXs in T2D therapy, might be attributed to the huge heterogeneity between studies in several aspects of study design [98,99]. Thus, there are variations between studies with regard to inclusion/exclusion criteria; in some studies, the target population was T2D patients, whereas in other studies included prediabetic but not diabetic patients [100,101,102]. In addition, studies vary with regard to the specific type of AOX given as well as the dosage and duration of supplementation. Moreover, many compounds, considered to act as AOXs, exert additional biological functions that make it difficult to isolate the anti-oxidative effect from other bioactivities of the compounds. For example, polyphenols are commonly used as AOX; however, these molecules might improve management of diabetes in various mechanisms that are not necessarily related to their antioxidative properties [103]. Thus, the improvement in some metabolic indices observed might be unrelated to neutralization of oxidative stress. In an effort to focus on the pure AOX effects, we will include in this discussion only studies of compounds with bioactivity which is mostly attributed to their function as AOX, such as vitamin C, E, selenium, the GPx1 mimetic ebselen, and N-acetylcysteine (NAC).

Unfortunately, Vitamins C and E were not found to prevent the onset of type 2 diabetes, as concluded from several large randomized controlled trials [104,105,106]. In addition, meta-analyses, and umbrella reviews of the use of these vitamins for the treatment of T2D, mostly agreed with the conclusion that these AOXs had only mild effects on glycemic control and on diabetic complications, such as cardiovascular and kidney diseases [107,108,109,110,111,112,113,114,115,116]. The effects of selenium on the prevalence of T2D were investigated as well. Selenium is incorporated into selenoproteins such as glutathione peroxidases and thioredoxin reductases, which are part of the AOX system. Unfortunately, selenium supplementation did not prevent T2D, and even increased the risk for the disease [117]. Ebselen also failed to reduce blood glucose and HbA1C in diabetic patients, however oxidative stress profile was not improved as well by this GPx1 mimetic [118]. Several clinical studies had also been performed regarding NAC, which exerts its AOX properties mainly by replenishing GSH pools and reducing disulfide bonds in proteins [119], and has an established safety profile [120]. Meta-analysis of NAC effects on diabetes-related cardiovascular complications did show some promising results [121], but effects of NAC on blood glucose levels have scarcely been reported, and the results are inconsistent (as described below).

With these disappointing data, obtained after several decades of research, there is a risk that the research of AOXs as potentially beneficial agents for the treatment of T2D would be abandoned. However, as stated by Malcolm Forbes “Failure is success if we learn from it”, we believe that by analyzing in depth the failed trials, there are several lessons that might be learned, and can direct as to a successful implementation of AOXs into the clinic.

## 5. Inappropriate Dosing as an Explanation for the Lack of Benefit

As mentioned, several explanations have been suggested for the disappointing results of AOXs supplementations in the prevention and treatment of T2D in human. In this review, we will focus on the ‘inappropriate dosing’ hypothesis, suggesting that with the lack of gold-standards for the diagnosis of oxidative stress in patients, either at baseline, during intervention, or at the end of the interventional course, AOXs are blindly given. Therefore, dose selection might be insufficient, leading to inadequate oxidative stress neutralization. Moreover, it might also be hypothesized, that with the lack of personalized evaluation of need, AOXs might be given in excess, especially when given as a preventive strategy, leading to undesirable depletion of ROS. In the next sections we will discuss these hypotheses.

### 5.1. Sub-Optimal Dosing of AOX as a Cause of Failure?

Clinical trials with the use of NAC at a maximal dose of 1200 mg/day, have demonstrated some efficacy in lowering diabetes-related complications such as high blood pressure and platelet-monocyte conjugation, while glycemic control was unaffected [122,123]. By comparing in vivo and clinical studies, we suggest that a NAC dose of 1200 mg/day might be less than require to be effective. Most in vivo studies, performed in rodents, demonstrated beneficial effects on the treatment of T2D and its complications upon administration of 600–1500 mg/kg/day of NAC [77,78,79,83,124]. By adapting mice to a human dose, using a metabolic conversion factor [125], the human effective dose should be 32–50 mg/kg/day, or 2.2–3.5 g/day for a 70 kg person. In accord with this, oral NAC administration of 600 or 1200 mg/day did not affect plasma thiol derivatives in healthy subjects [126]. Similarly, it was demonstrated that NAC alters cysteine- and glutathione-based thiols in a dose-dependent manner, showing an increase with 35 mg/kg/day and a maximal effect at 70 mg/kg/day (2.5 and 5 g, respectively, for a 70 kg person) [127]. These doses equal the above-mentioned effective calculated dose and suggest a simple explanation for the failure of studies using sub-optimal doses of NAC.

Moreover, the effective dose, for the alleviation of glucose intolerance and insulin resistance, must be adjusted to the target population. In mice, an effective NAC dose was different among research models; doses required to introduce an improvement in glucose tolerance in mice models of diabetes were higher than those required in glucose-intolerance models [77,83,124,128]. In support of the importance of a personalized AOX administration, it was reported that in non-obese, non-diabetic PCOS patients, NAC improved insulin sensitivity when given at 1.8 gr/day [100,101,129], whereas a higher dose (3 gr/day) and a longer time of treatment were required in obese patients [101]. In addition, it was recently reported that NAC at a dose of 1.8 gr/day improved insulin sensitivity in non-diabetic MetS patients [130]. Thus, by increasing the NAC dose to 1.8 gr/day, insulin sensitivity appears to be significantly improved in non-diabetic individuals. It is expected that an even higher dose would be required to improve indices of diabetes in patients with overt type 2 diabetes. Although clinical data on NAC supplementation to T2D patients are lacking, a recent pilot study showed that a combination of a high dose of NAC and glycine (7 gr/day each, for a 70 kg person) improved insulin sensitivity in poorly-controlled T2D patients, presumably by restoration of GSH pools [102].

Therefore, we assume that one of the possible explanations for the minimal beneficial outcomes of AOX therapy in diabetes is simply the use of sub-optimal doses. This hypothesis might explain the failure of some clinical studies utilizing NAC as an anti-diabetic therapy, but presumably might also be implicated for other AOXs.

### 5.2. Can Glycemic Control Be Adversely Affected by Excess AOXs?

In spite of their destructive potential, it is well-known that physiological levels of ROS are required for several cell functions [131] and that controlled changes in ROS concentration temporarily alter the redox state, enabling redox signaling and regulation of various cellular processes [4]. Accordingly, it might further be hypothesized that excess AOX consumption may adversely affect health as a result of ROS depletion. In the following sections, we will describe principles of redox signaling, the role of ROS in insulin signaling and insulin secretion, and we will discuss the clinical relevance of these observations.

#### 5.2.1. Redox Regulation of Proteins and Cell Signaling

Redox signaling involves reversible oxidative modifications of proteins. Certain cysteine thiol groups (-SH) of proteins are maintained in a reduced state by default, and the oxidative environment allows these thiols to become temporarily oxidized to the form of sulfenic acid [132,133]. Sulfenic acid is an unstable intermediate for the generation of disulfide bonds (S-S) which may be formed between either protein subunits or different domains of the same protein, thus altering conformation and activity. Additionally, sulfenic acid is an intermediate state for the glutathionylation of the thiol (S-SG) residue for the generation of mixed disulfides [134,135,136]. Protein glutathionylation is a reversible post-translational modification that can either increase or decrease activity of proteins [137,138].

Reversible oxidative modifications of signaling proteins play a role in regulating cell signaling, somewhat reminiscent of the well-characterized regulation by reversible phosphorylation/dephosphorylation [136,139]. However, reversible oxidative modifications might be oxidized further, leading to irreversible protein modification, which usually results in protein dysfunction and degradation and/or the formation of dysfunctional protein aggregates [136]. Thus, the protein cysteine residue can be reversibly oxidized to sulfenic acid (SOH), which is a physiological event and an intermediate step in the generation of disulfide bonds or the glutathionylation of proteins [136]. These kinds of modifications affect protein–protein interactions and protein function and thus are important players in regulating cell signaling. However, under oxidized conditions, sulfenic acid might be irreversibly further oxidized to sulfinic (SO_2_H) and sulfonic acids (SO_3_H), leading to protein misfolding, aggregation, and degradation. This scenario demonstrates the necessity for tight regulation of redox homeostasis to enable delicate and temporal redox balance shifts for the transmission of redox signaling, thus preventing chronic, supra-physiological levels of ROS that generate oxidative stress.

Many cellular processes, such as cell cycle regulation, cellular growth, and protein phosphorylation/dephosphorylation, are regulated by redox signaling [39]. Signaling proteins, which are found to be a target of redox regulation, include transcription factors (i.e., AP-1, Nrf2 and NF-_K_B), kinases (Src family kinases, MAP kinases), as well as protein tyrosine phosphatases (PTPs) [4].

#### 5.2.2. Redox Regulation of Insulin Signaling

Accumulating evidence suggests the modulation of insulin signaling by ROS, as was reviewed previously [140,141]. This effect seems to be dependent upon both ROS concentration and the duration of exposure [40,142]. H_2_O_2_ in particular has been reported to influence several physiological functions of insulin, such as glucose transport, glycogen synthesis, lipogenesis and regulation of gene expression [16,40,143,144]. Furthermore, insulin stimulation of cells was found to be accompanied by sulfhydryl oxidation and the generation of small amounts of H_2_O_2_ via the NADPH oxidase (NOX)-dependent system [40,142,145]. NOX inhibition abolished the insulin-dependent phosphorylation of IR and IRS1, indicating that insulin-induced generation of H_2_O_2_ is necessary for transmission of the cascade. Thus, insulin itself provokes the generation of ROS, which play a role as second messengers in insulin signaling [146,147,148].

Tyrosine phosphorylation of insulin receptor (IR) and its substrate (IRS) is crucial for the initiation and transmission of insulin signaling, leading to a cascade of protein phosphorylations that mediate the metabolic effects of the hormone. In contrast, phosphatases dephosphorylate and—for the most part—negatively regulate the insulin signaling cascade. It was shown that both kinases and phosphatases are regulated by oxidative modifications [4,147,149,150]. The kinase activity of IR itself is modulated by H_2_O_2_ through the oxidation of Cys1245–Cys1308, leading to an alteration in ATP binding, which is required for the auto-phosphorylation of the receptor [149]. Another interesting mechanism that might link redox balance to the capability to activate insulin receptors is through the glutathionylation of galectin 3. Galectin 3 is a member of the β-galactoside-binding lectin family, mainly produced by immune cells, and was found to be elevated in T2D patients. Direct binding of galectin 3 to insulin receptor decreases autophosphorylation and activation of the receptor, leading to attenuation of insulin-induced glucose transport [151]. Interestingly, a recent publication demonstrated that galectin 3 is prone to glutathionylation (Cys 187), an event that eliminates its interaction with the receptor [152]. Whereas a moderate oxidative environment promotes galectin 3 glutathionylation, this process is abolished under conditions of both oxidative and reductive stress, further emphasizing the effects of redox imbalance on IR activation.

**Figure 2:**
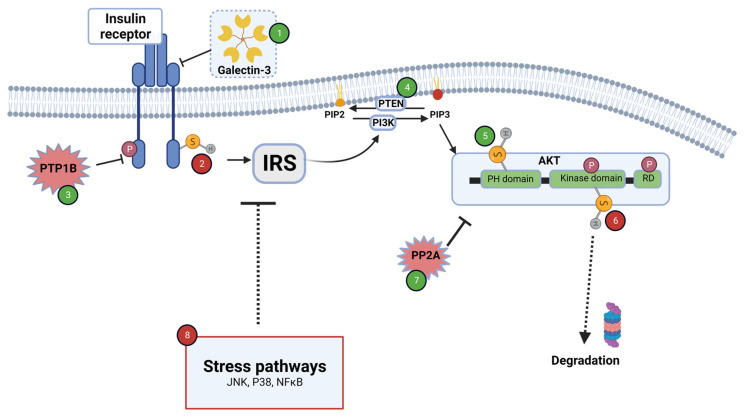
Redox regulation of insulin signaling in target tissues of insulin action (i.e., muscle, adipose tissue and liver). ROS exert positive and negative effects on insulin signaling. Stimulatory events are marked in green numbers, while inhibitory events are marked in red numbers. ROS-dependent stimulatory mechanisms: The inhibitory effect of Galectin-3 on insulin receptor is prevented upon glutathionylation of the lectin (1). PTP1B is inhibited by oxidation of critical thiols located in catalytic area, enabling stimulation of insulin signaling (3). PTEN is inhibited by oxidation, thus abolishing PIP3 dephosphorylation (4). Oxidation of thiol residue in the PH domain of Akt enhances its binding affinity to PIP3 (5). Oxidation of PTP2A diminishes its activity, thus preserving phosphorylation and activation of Akt (7). ROS-dependent inhibitory mechanisms: Oxidation of thiol groups (Cys1245–Cys1308) located on the intracellular domain of insulin receptor abrogates ATP binding and receptor activation (2). Proteasomal degradation of Akt is stimulated upon oxidation of Cys in kinase domain (6). ROS might activate stress pathways such as Jnk and p38, leading to an increase in IRS serine phosphorylation, associated with insulin resistance (8). See details in text. Created with BioRender.com (accessed on 5 March 2023).

Physiological concentrations of H_2_O_2_ inhibit the tyrosine phosphatase PTP1B by oxidizing thiol residues located in the catalytic area of the enzyme. Inactivation of PTP1B leads to elevated basal tyrosine phosphorylation of both IR and IRS, thereby enhancing insulin signaling [40,142,147,153]. Other phosphatases, such as mitogen-activated protein (MAP) kinase phosphatase 1 [154] and phosphatase, and tensin homolog deleted on chromosome 10 (PTEN), are also found to be important targets for redox regulation, the process being inhibited by oxidation [147,154,155]. PTEN dephosphorylates phosphoinositol triphosphate (PIP3), which is required for the recruitment of Akt to the plasma membrane and for its activation. Akt is an important Ser/Thr kinase, mediating insulin action. While insulin-induced activation of PI3K increases PIP3 levels, PTEN dephosphorylates this lipid, leading to the attenuation of insulin signaling [147]. Thus, by oxidation of PTEN and inhibition of its activity, transmission of insulin signaling is enhanced. In addition, the dephosphorylation of Akt is mediated by Ser/Thr protein phosphatase 2A (PP2A), which is inhibited by oxidation as well [156]. Thus, the oxidative reaction inhibits the activity of a set of phosphatases, leading to augmentation of insulin signaling. Moreover, Akt by itself is reversibly oxidized on cysteine residues located in PH domains, leading to an increased binding affinity to PIP3 [157]. Interestingly, oxidation of thiol residues, located in the kinase domain, reduces Akt activity and elevates its degradation in the proteasome, indicating the presence of highly specific machinery that regulates thiol oxidation in a site-specific manner [154,157]. The positive and negative effects of ROS on insulin signaling are illustrated in Figure 2.

Finally, oxidants are also required for protein folding in the ER, and the lack of oxidants will lead to ER stress and stimulation of the unfolded-protein response, which alters insulin sensitivity in muscle and liver [158,159].

#### 5.2.3. ROS Depletion and Insulin Signaling

Since ROS are necessary for insulin signaling, a lack of these species might be expected to attenuate the transmission of the signal. In fact, ROS depletion may promote insulin resistance. Overexpression of the AOX enzyme Gpx1 led to insulin resistance and hyperglycemia in mice, whereas a deficiency of Gpx1 may actually protect mice from obesity-induced insulin resistance [160,161,162]. Furthermore, an increase in the levels of Selenoprotein P, which is responsible for the transport of selenium from liver to other tissues, was reported to be associated with increased risk of T2D [163]. This adverse effect is suggested to be mediated by impaired insulin signaling and dysregulated glucose metabolism in hepatocytes and myocytes, presumably as a result of a reductive stress [163,164]. In addition, we showed that NAC inhibited insulin-stimulated glucose uptake in adipocytes. In addition, insulin-induced phosphorylation of signaling proteins was diminished in NAC-treated myotubes and adipocytes [143]. NAC is a precursor of glutathione; thus, the effects of elevated glutathione levels on insulin signaling is of interest. NAC increased levels of glutathione and free thiols in myotubes and adipocytes and reduced the extent of protein glutathionylation [143]. Among these proteins, we found galectin 3 to be deglutathionylated, suggesting a putative mechanism for the inhibition of insulin signaling by NAC, through the induction of galectin 3-insulin receptor binding.

In additional studies, it was reported that GSH inhibited insulin-dependent IRS-1 and Akt phosphorylation in 3T3-L1 adipocytes [165], presumably resulting from a reduction in disulfide bonds in certain proteins [166]. Furthermore, GSH levels were found to be elevated in adipose tissue of diabetic ob/ob mice [165], and glutathionylated proteins were reduced in obese compared to lean rats [167]. These reports indicate that excessive levels of reduced species may be as detrimental as oxidative stress and could be implicated in insulin resistance.

While most in vivo studies have demonstrated the benefits of AOX supplementation in reducing the severity of glucose intolerance and insulin resistance, it was also shown that excess AOXs negatively affect insulin sensitivity in rodents. An inverted U-shaped curve of insulin sensitivity was reported in NAC-treated T2D mice, demonstrating adverse effects of high-dose NAC on insulin signaling [124]. In addition, administration of NAC to normo-glycemic C57bl mice caused a reduction in insulin-induced phosphorylation of Akt, GSK3β and PRAS40, even though other parameters, such as blood glucose levels, were unaffected [143]. Similarly, chronic administration of vitamin C and E to normo-glycemic, healthy rats impaired glucose tolerance and insulin sensitivity, effects that were well-correlated with an increase in hepatic GSH in these animals [168]. In addition, there are reports showing that by neutralizing ROS generated during physical activity, AOXs eliminate beneficial metabolic outcomes of physical activity and enhance the risk of developing insulin resistance [169,170,171,172]. These data indicate that excessive AOXs impair insulin signaling and further emphasize the deleterious consequences of an unbalanced redox shift to the reduced side, leading to a “reductive stress”.

Thus, reductive stress, achieved by genetic manipulation of AOX enzymes or by AOX treatment, may lead to harmful effects on insulin action. However, since these observations were obtained from in vitro and in vivo experiments, the clinical relevance of these findings must be validated. Evidence of adverse effects of AOX consumption on insulin sensitivity in humans is scarce and mostly documented for selenium. These studies report a U-shaped therapeutic dose effect for selenium, with a narrow window of beneficial dose [173] and an increased risk of T2D with elevated selenium levels [117,174,175].

#### 5.2.4. Redox Regulation of Insulin Secretion

Pancreatic β-cells maintain blood glucose levels by secreting insulin in response to increased glucose concentration in the blood. The mechanism of insulin secretion is stimulated by glucose entry to β-cells, facilitated by glucose transporter 2 (GLUT2). Glucose is then metabolized, leading to an elevated ATP/ADP ratio and closure of ATP-sensitive K^+^ channels. The resulting depolarization stimulates the opening of voltage-dependent Ca^2+^ channels and influx of Ca^2+^, thereby triggering exocytosis of insulin-containing vesicles [176].

On the one hand, the deleterious effects of oxidative stress on pancreatic beta-cells are well known [26]. On the other hand, the role of physiological levels of ROS in regulating beta-cell function is controversial [177,178], with ROS suggested to be either positive or negative regulators of insulin secretion [176]. There is evidence demonstrating that ROS enhances glucose-induced insulin secretion [177,179], and it was further suggested that glucose-induced activation of NOX, leading to the generation of ROS, is actually required for insulin secretion [180,181]. However, many other studies demonstrated completely opposing observations, showing that physiological levels of ROS reduce insulin secretion. Thus, H_2_O_2_ reportedly stimulates the activity of ATP-sensitive K^+^ channels, leading to cell hyperpolarization and depression of insulin secretion [182]. In addition, low-dose H_2_O_2_ inhibited glucose-induced insulin secretion in isolated rat pancreatic islets [178]. Moreover, glucose reduces ROS levels in a dose-dependent manner [183], thus enabling proper secretion of insulin. We also found that low concentrations of 5 μM H_2_O_2_, which is within the physiological range, inhibited insulin secretion under low-glucose conditions, while insulin secretion, stimulated by high-glucose levels, was not impaired [184]. Cell death was observed only with higher doses of H_2_O_2_ (100 μM), suggesting that the negative effect of H_2_O_2_ on insulin secretion is mediated by redox regulation of the process, rather than an outcome of oxidative stress damage. Finally, in support of a negative correlation between ROS and insulin secretion, it was reported that HFD feeding reduced NOX expression in rat pancreatic islets, leading to diminished ROS production and elevated insulin secretion [181].

The inhibitory effect of H_2_O_2_ on insulin secretion is presumably mediated by the attenuation of glucose oxidation, perhaps through the inhibition of glyceraldehyde-3-phosphate-dehydrogenase and aconitase [178,185,186]. Slowing down the rate of glucose metabolism decreased the ATP/ADP ratio and downstream events required for insulin secretion. Thus, a negative correlation was observed between ROS levels in beta cells and insulin secretion. It was suggested that glucose activates a fast response of increased AOX capacity for the reduction of ROS. It was shown that glucose actually increased the GSH/GSSG ratio, in parallel with an increase in the NADPH/NADP^+^ ratio [183,187]. The fast AOX response includes activation of SOD and the NADPH-generating pentose-phosphate pathway, leading to reduced ROS levels and proper insulin secretion [188,189].

Therefore, most studies conclude that physiological concentrations of ROS are involved in the negative regulation of insulin secretion. This is mediated by redox signaling and is a transitory and reversible event. As glucose levels rise, ROS levels are reduced, and insulin secretion increases. In contrast to physiological conditions, chronic hyperglycemia leads to a persistent pathological increase in ROS associated with oxidative stress and accumulating damage to beta cells, leading to their dysfunction and death.

The controversy among the various studies regarding stimulatory or inhibitory effects of ROS on insulin secretion represents the complexity of redox signaling leading to the dual impact of ROS, found to be either positive or negative regulators of insulin secretion. These opposing effects might depend on nutrient regulation and the metabolic state of the cells as well as on the subcellular compartmentation of ROS [176,190,191].

#### 5.2.5. ROS Depletion and Insulin Secretion

As discussed above, there is disagreement regarding the role of ROS in the regulation of insulin secretion. With most studies demonstrating the inhibitory effect of ROS, it might be expected that neutralizing ROS would antagonize the ROS effect and improve insulin secretion.

NOX inhibition, achieved either by genetic manipulation or pharmacological intervention, leads to elevated insulin secretion [192,193]. Similar results were obtained by increasing the activity of the antioxidant system, either by administration of PEG-catalase or by Gpx1 overexpression, both of which preserve the function and survival of the islets [84,189]. Potential benefits were also observed with vitamin E. Derivatives of vitamin E (tocotrienols) potentiated insulin secretion in rat islets [194]. In GK rats, a model of non-obese T2D, it was reported that chronic hyperglycemia induces oxidative stress in pancreatic β-cells. Vitamin E administration to these rats increased insulin secretion, leading to improved glucose tolerance and lower HbA1c levels [195].

NAC has also been investigated for its effects on pancreatic beta cells. NAC improved glucose tolerance and prevented hyperinsulinemia in diabetic db/db mice, while islets mass was increased, presumably resulting from blockade of apoptosis [196]. In HFD-fed mice, NAC improved insulin sensitivity, an effect that prevents compensatory hypertrophy of islets and resulting hyperglycemia and avoids HFD-induced loss of beta-cell identity. It is difficult, however, to differentiate between direct benefits of NAC on pancreatic islets function and those from its insulin-sensitizing properties [197]. Therefore, conclusions regarding effects of NAC and other AOXs on pancreatic islets’ functionality and their survival are mostly derived from in vitro and ex vivo experiments. In these studies, it was reported that insulin secretion was indeed elevated in isolated islets of NAC-treated diabetic KK-Ay mice [143] as well as in NAC-treated Zucker diabetic rats [79]. When given to isolated islets, NAC neutralized oxidative stress and restored insulin secretion in oxidatively stressed dexamethasone-treated islets [198]. However, in islets of normo-glycemic ICR mice, NAC did not exert any positive or negative effects on pancreatic beta-cell function or viability, even at millimolar concentrations which were found to disturb insulin action in adipocytes and myotubes [143]. The absence of negative outcomes of NAC-dependent reductive stress in these islets indicates that, whereas pancreatic islets are highly vulnerable to oxidative shifts, they are not much affected by reductive stress. Therefore, NAC appears to possess the capability to neutralize oxidative stress and to improve function in oxidative stressed islets but does not impair islets’ function even when given in excess.

## 6. Summary and Clinical Implications

In this review, we have discussed the effects of ROS on glucose balance in both physiological conditions and in situations of ROS accumulation to pathological levels, namely oxidative stress. Oxidative stress has detrimental effects on all systems involved in glucose homeostasis, including insulin-responsive tissues and in insulin-secreting cells. Conversely, physiological levels of ROS regulate certain signaling pathways and might be positive or negative regulators of cellular functions in a tissue-specific manner. Accordingly, and considering ROS action, AOXs may be beneficial for both neutralization of oxidative stress and prevention of its deleterious consequences, but might also exert negative effects if administered unnecessarily as a result of an excessive ROS depletion.

**Figure 3:**
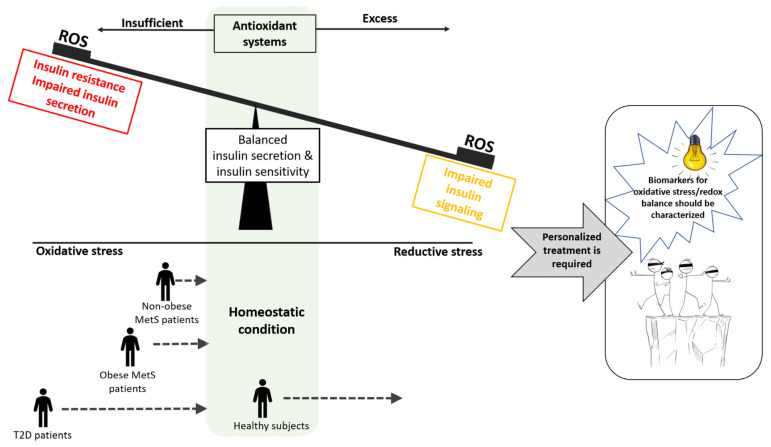
Inappropriate dosing of AOXs may contribute to the failure of these agents in clinical studies. Homeostatic redox balance is required to preserve an optimal insulin secretion and insulin sensitivity. Insufficient AOX activity leads to oxidative stress that promotes development of T2D. It is assumed that elevating severities of oxidative stress is presented in patients with MetS, obesity and overt T2D. AOX dose should be adapted to severity of oxidative stress. On the other hand, excess AOXs might deplete ROS, leading to a reductive stress, which was shown to impair insulin signaling. Therefore, AOX supplementations given as a preventive strategy to a healthy subject might lead to adverse outcomes. Biomarkers for accurate measurement of oxidative stress should be developed to optimize AOX benefits.

With this in mind, we may conclude that AOX supplementation as a strategy to prevent onset of T2D in the absence of any indices for the presence of oxidative stress would not be recommended and might even impair insulin sensitivity. With regard to therapy of an overt disease, since T2D is coupled to the presence of oxidative stress, we may assume that patients might benefit from AOX therapy. However, the efficiency of AOX treatment should be significantly optimized by adjusting dosage to the severity of oxidative stress (see Figure 3). For this approach, biomarkers to be used as measures of the need and the dose should be defined in order to be sufficient—but not excessive—to neutralize oxidative stress. Unfortunately, measurement of oxidative stress is challenging. It might be evaluated by analyzing the ratio between oxidized/reduced molecules (i.e., GSH/GSSG), and the level of AOXs or markers of oxidative stress-induced damage (i.e., MDA and protein carbonyls). With the understanding that oxidative stress may not be properly measured by a single biomarker, several approaches have been used to establish an oxidative stress score. This issue is not in the scope of this review and is covered by several recent publications [199,200]. However, there is still no accepted gold-standard score, which is a major obstacle in the path towards optimizing the therapeutic potential of AOXs.

## 7. Conclusions

In order to optimize its benefits, AOX therapy should be given in a personalized manner according to an individual assessment which includes the presence and severity of oxidative stress. Unfortunately, while oxidative stress is a well-recognized pathological factor, clear clinical definitions for its diagnosis are missing, and no gold-standard biomarkers for the quantification of this alteration have been defined. Thus, AOXs are blindly consumed without an examination of their necessity. Furthermore, with the absence of gold standards for the detection of oxidative stress, one cannot know whether treatment goals might have been achieved according to redox balance. Therefore, until accurate measures of OS are characterized, AOX to prevent ROS depletion should definitely not be recommended as a preventive strategy for T2D. With regard to the treatment of overt diabetes, we believe that the current failure of AOX to improve indices of diabetes might be simply related to the insufficient neutralization of oxidative stress. With the development of OS biomarkers, optimization of AOXs therapy should be determined to maximize the therapeutic potential of these agents.

## References

[1] Gough D.R., Cotter T.G. (2011). Hydrogen peroxide: A Jekyll and Hyde signalling molecule. Cell Death Dis..

[2] Zhang L., Wang X., Cueto R., Effi C., Zhang Y., Tan H., Qin X., Ji Y., Yang X., Wang H. (2019). Biochemical basis and metabolic interplay of redox regulation. Redox Biol..

[3] Brandes R.P., Rezende F., Schroder K. (2018). Redox Regulation Beyond ROS: Why ROS Should Not Be Measured as Often. Circ. Res..

[4] Valko M., Leibfritz D., Moncol J., Cronin M.T., Mazur M., Telser J. (2007). Free radicals and antioxidants in normal physiological functions and human disease. Int. J. Biochem. Cell Biol..

[5] Bae Y.S., Oh H., Rhee S.G., Yoo Y.D. (2011). Regulation of reactive oxygen species generation in cell signaling. Mol. Cells.

[6] Harman D. (1984). Free radical theory of aging: The “free radical” diseases. AGE.

[7] Ghosh N., Chatterjee S., Sil P.C., Nabavi S.M., Silva A.S. (2022). Chapter1.1—Evolution of antioxidants over times (including current global market and trend). Antioxidants Effects in Health.

[8] Ziegler M., Wallert M., Lorkowski S., Peter K. (2020). Cardiovascular and Metabolic Protection by Vitamin E: A Matter of Treatment Strategy?. Antioxidants.

[9] Giannopoulos G., Angelidis C., Vogiatzi G., Cleman M.W., Deftereos S. (2018). Antioxidant treatment in peripheral artery disease: The rationale is there, but what about clinical results?. Curr. Opin. Pharmacol..

[10] Thomson N.C. (2018). Targeting oxidant-dependent mechanisms for the treatment of respiratory diseases and their comorbidities. Curr. Opin. Pharmacol..

[11] Mariano C. (2005). Review: High dose vitamin E supplementation is associated with increased all cause mortality. Evid. Based Nurs..

[12] Sies H., Berndt C., Jones D.P. (2017). Oxidative Stress. Annu. Rev. Biochem..

[13] Ursini F., Maiorino M., Forman H.J. (2016). Redox homeostasis: The Golden Mean of healthy living. Redox Biol..

[14] Xie J., Wang M., Long Z., Ning H., Li J., Cao Y., Liao Y., Liu G., Wang F., Pan A. (2022). Global burden of type 2 diabetes in adolescents and young adults, 1990-2019: Systematic analysis of the Global Burden of Disease Study 2019. BMJ.

[15] Bhatti J.S., Sehrawat A., Mishra J., Sidhu I.S., Navik U., Khullar N., Kumar S., Bhatti G.K., Reddy P.H. (2022). Oxidative stress in the pathophysiology of type 2 diabetes and related complications: Current therapeutics strategies and future perspectives. Free Radic. Biol. Med..

[16] Bashan N., Kovsan J., Kachko I., Ovadia H., Rudich A. (2009). Positive and negative regulation of insulin signaling by reactive oxygen and nitrogen species. Physiol. Rev..

[17] Baynes J.W. (1991). Role of oxidative stress in development of complications in diabetes. Diabetes.

[18] Monnier L., Mas E., Ginet C., Michel F., Villon L., Cristol J.P., Colette C. (2006). Activation of oxidative stress by acute glucose fluctuations compared with sustained chronic hyperglycemia in patients with type 2 diabetes. JAMA.

[19] Monserrat-Mesquida M., Quetglas-Llabrés M., Capó X., Bouzas C., Mateos D., Pons A., Tur J.A., Sureda A. (2020). Metabolic Syndrome is Associated with Oxidative Stress and Proinflammatory State. Antioxidants.

[20] Meigs J.B., Larson M.G., Fox C.S., Keaney J.F., Vasan R.S., Benjamin E.J. (2007). Association of oxidative stress, insulin resistance, and diabetes risk phenotypes: The Framingham Offspring Study. Diabetes Care.

[21] Chen L., Xu W.M., Zhang D. (2014). Association of abdominal obesity, insulin resistance, and oxidative stress in adipose tissue in women with polycystic ovary syndrome. Fertil. Steril..

[22] Furukawa S., Fujita T., Shimabukuro M., Iwaki M., Yamada Y., Nakajima Y., Nakayama O., Makishima M., Matsuda M., Shimomura I. (2004). Increased oxidative stress in obesity and its impact on metabolic syndrome. J. Clin. Investig..

[23] Gumieniczek A., Hopkala H., Wojtowicz Z., Nieradko M. (2001). Differences in antioxidant status in skeletal muscle tissue in experimental diabetes. Clin. Chim. Acta.

[24] Houstis N., Rosen E.D., Lander E.S. (2006). Reactive oxygen species have a causal role in multiple forms of insulin resistance. Nature.

[25] Robertson R., Zhou H., Zhang T., Harmon J.S. (2007). Chronic oxidative stress as a mechanism for glucose toxicity of the beta cell in type 2 diabetes. Cell Biochem. Biophys..

[26] Robertson R.P. (2004). Chronic oxidative stress as a central mechanism for glucose toxicity in pancreatic islet beta cells in diabetes. J. Biol. Chem..

[27] Kajimoto Y., Kaneto H., Lee H.K., DiMauro S., Tanaka M., Wei Y.-H. (2004). Role of Oxidative Stress in Pancreatic β-Cell Dysfunction. Mitochondrial Pathogenesis: From Genes and Apoptosis to Aging and Disease.

[28] Burgos-Moron E., Abad-Jimenez Z., Maranon A.M., Iannantuoni F., Escribano-Lopez I., Lopez-Domenech S., Salom C., Jover A., Mora V., Roldan I. (2019). Relationship between Oxidative Stress, ER Stress, and Inflammation in Type 2 Diabetes: The Battle Continues. J. Clin. Med..

[29] Townsend D.M. (2007). S-glutathionylation: Indicator of cell stress and regulator of the unfolded protein response. Mol. Interv..

[30] Szaleczky E., Prechl J., Fehér J., Somogyi A. (1999). Alterations in enzymatic antioxidant defence in diabetes mellitus--a rational approach. Postgrad. Med. J..

[31] Gawlik K., Naskalski J.W., Fedak D., Pawlica-Gosiewska D., Grudzień U., Dumnicka P., Małecki M.T., Solnica B. (2016). Markers of Antioxidant Defense in Patients with Type 2 Diabetes. Oxid. Med. Cell Longev..

[32] Livingstone C., Davis J. (2007). Review: Targeting therapeutics against glutathione depletion in diabetes and its complications. Br. J. Diabetes Vasc. Dis..

[33] Lagman M., Ly J., Saing T., Kaur Singh M., Vera Tudela E., Morris D., Chi P.T., Ochoa C., Sathananthan A., Venketaraman V. (2015). Investigating the causes for decreased levels of glutathione in individuals with type II diabetes. PLoS ONE.

[34] Chung S.S., Ho E.C., Lam K.S., Chung S.K. (2003). Contribution of polyol pathway to diabetes-induced oxidative stress. J. Am. Soc. Nephrol..

[35] Sekhar R.V., McKay S.V., Patel S.G., Guthikonda A.P., Reddy V.T., Balasubramanyam A., Jahoor F. (2011). Glutathione synthesis is diminished in patients with uncontrolled diabetes and restored by dietary supplementation with cysteine and glycine. Diabetes Care.

[36] Lutchmansingh F.K., Hsu J.W., Bennett F.I., Badaloo A.V., McFarlane-Anderson N., Gordon-Strachan G.M., Wright-Pascoe R.A., Jahoor F., Boyne M.S. (2018). Glutathione metabolism in type 2 diabetes and its relationship with microvascular complications and glycemia. PLoS ONE.

[37] Chang Y.C., Chuang L.M. (2010). The role of oxidative stress in the pathogenesis of type 2 diabetes: From molecular mechanism to clinical implication. Am. J. Transl. Res..

[38] Roberts C.K., Sindhu K.K. (2009). Oxidative stress and metabolic syndrome. Life Sci..

[39] Rains J.L., Jain S.K. (2011). Oxidative stress, insulin signaling, and diabetes. Free Radic. Biol. Med..

[40] Srivastava A.K. (2005). Redox regulation of insulin action and signaling. Antioxid. Redox Signal..

[41] Rudich A., Tirosh A., Potashnik R., Hemi R., Kanety H., Bashan N. (1998). Prolonged oxidative stress impairs insulin-induced GLUT4 translocation in 3T3-L1 adipocytes. Diabetes.

[42] Archuleta T.L., Lemieux A.M., Saengsirisuwan V., Teachey M.K., Lindborg K.A., Kim J.S., Henriksen E.J. (2009). Oxidant stress-induced loss of IRS-1 and IRS-2 proteins in rat skeletal muscle: Role of p38 MAPK. Free Radic. Biol. Med..

[43] Kamata H., Honda S., Maeda S., Chang L., Hirata H., Karin M. (2005). Reactive oxygen species promote TNFalpha-induced death and sustained JNK activation by inhibiting MAP kinase phosphatases. Cell.

[44] Zhou J., Xu G., Bai Z., Li K., Yan J., Li F., Ma S., Xu H., Huang K. (2015). Selenite exacerbates hepatic insulin resistance in mouse model of type 2 diabetes through oxidative stress-mediated JNK pathway. Toxicol. Appl. Pharmacol..

[45] Ogihara T., Asano T., Katagiri H., Sakoda H., Anai M., Shojima N., Ono H., Fujishiro M., Kushiyama A., Fukushima Y. (2004). Oxidative stress induces insulin resistance by activating the nuclear factor-kappa B pathway and disrupting normal subcellular distribution of phosphatidylinositol 3-kinase. Diabetologia.

[46] Chiu W.C., Chen C.J., Lee T.S., Chen Z.J., Ke P.H., Chiang A.N. (2010). Oxidative stress enhances AP-1 and NF-kappaB-mediated regulation of beta(2)-glycoprotein I gene expression in hepatoma cells. J. Cell Biochem..

[47] Pessler D., Rudich A., Bashan N. (2001). Oxidative stress impairs nuclear proteins binding to the insulin responsive element in the GLUT4 promoter. Diabetologia.

[48] Bhatti J.S., Bhatti G.K., Reddy P.H. (2017). Mitochondrial dysfunction and oxidative stress in metabolic disorders—A step towards mitochondria based therapeutic strategies. Biochim. Biophys. Acta BBA Mol. Basis Dis..

[49] Kelley D.E., He J., Menshikova E.V., Ritov V.B. (2002). Dysfunction of mitochondria in human skeletal muscle in type 2 diabetes. Diabetes.

[50] Schrauwen-Hinderling V.B., Kooi M.E., Hesselink M.K., Jeneson J.A., Backes W.H., van Echteld C.J., van Engelshoven J.M., Mensink M., Schrauwen P. (2007). Impaired in vivo mitochondrial function but similar intramyocellular lipid content in patients with type 2 diabetes mellitus and BMI-matched control subjects. Diabetologia.

[51] Sergi D., Naumovski N., Heilbronn L.K., Abeywardena M., O’Callaghan N., Lionetti L., Luscombe-Marsh N. (2019). Mitochondrial (Dys)function and Insulin Resistance: From Pathophysiological Molecular Mechanisms to the Impact of Diet. Front. Physiol..

[52] Kelley D.E., Mandarino L.J. (2000). Fuel selection in human skeletal muscle in insulin resistance: A reexamination. Diabetes.

[53] Donath M.Y., Ehses J.A., Maedler K., Schumann D.M., Ellingsgaard H., Eppler E., Reinecke M. (2005). Mechanisms of beta-cell death in type 2 diabetes. Diabetes.

[54] Karunakaran U., Park K.G. (2013). A systematic review of oxidative stress and safety of antioxidants in diabetes: Focus on islets and their defense. Diabetes Metab. J..

[55] Lenzen S. (2008). Oxidative stress: The vulnerable beta-cell. Biochem. Soc. Trans..

[56] Kajimoto Y., Kaneto H. (2004). Role of oxidative stress in pancreatic beta-cell dysfunction. Ann. N. Y. Acad. Sci..

[57] Kawamori D., Kajimoto Y., Kaneto H., Umayahara Y., Fujitani Y., Miyatsuka T., Watada H., Leibiger I.B., Yamasaki Y., Hori M. (2003). Oxidative stress induces nucleo-cytoplasmic translocation of pancreatic transcription factor PDX-1 through activation of c-Jun NH(2)-terminal kinase. Diabetes.

[58] Kaneto H., Kawamori D., Matsuoka T.A., Kajimoto Y., Yamasaki Y. (2005). Oxidative stress and pancreatic beta-cell dysfunction. Am. J. Ther..

[59] Yuan H., Zhang X., Huang X., Lu Y., Tang W., Man Y., Wang S., Xi J., Li J. (2010). NADPH oxidase 2-derived reactive oxygen species mediate FFAs-induced dysfunction and apoptosis of beta-cells via JNK, p38 MAPK and p53 pathways. PLoS ONE.

[60] Hou N., Torii S., Saito N., Hosaka M., Takeuchi T. (2008). Reactive oxygen species-mediated pancreatic beta-cell death is regulated by interactions between stress-activated protein kinases, p38 and c-Jun N-terminal kinase, and mitogen-activated protein kinase phosphatases. Endocrinology.

[61] Giacco F., Brownlee M. (2010). Oxidative stress and diabetic complications. Circ. Res..

[62] Forbes J.M., Cooper M.E. (2013). Mechanisms of diabetic complications. Physiol. Rev..

[63] Tossounian M.-A., Zhang B., Gout I. (2020). The Writers, Readers, and Erasers in Redox Regulation of GAPDH. Antioxidants.

[64] Yao D., Brownlee M. (2010). Hyperglycemia-induced reactive oxygen species increase expression of the receptor for advanced glycation end products (RAGE) and RAGE ligands. Diabetes.

[65] Du X., Matsumura T., Edelstein D., Rossetti L., Zsengeller Z., Szabo C., Brownlee M. (2003). Inhibition of GAPDH activity by poly(ADP-ribose) polymerase activates three major pathways of hyperglycemic damage in endothelial cells. J. Clin. Investig..

[66] Twarda-Clapa A., Olczak A., Białkowska A.M., Koziołkiewicz M. (2022). Advanced Glycation End-Products (AGEs): Formation, Chemistry, Classification, Receptors, and Diseases Related to AGEs. Cells.

[67] Iacobini C., Vitale M., Pesce C., Pugliese G., Menini S. (2021). Diabetic Complications and Oxidative Stress: A 20-Year Voyage Back in Time and Back to the Future. Antioxidants.

[68] Lien C.F., Chen S.J., Tsai M.C., Lin C.S. (2021). Potential Role of Protein Kinase C in the Pathophysiology of Diabetes-Associated Atherosclerosis. Front. Pharmacol..

[69] Zhao S., Cheng C.K., Zhang C.L., Huang Y. (2021). Interplay Between Oxidative Stress, Cyclooxygenases, and Prostanoids in Cardiovascular Diseases. Antioxid. Redox Signal..

[70] Bai Y., Ma J.X., Guo J., Wang J., Zhu M., Chen Y., Le Y.Z. (2009). Muller cell-derived VEGF is a significant contributor to retinal neovascularization. J. Pathol..

[71] Rossino M.G., Lulli M., Amato R., Cammalleri M., Monte M.D., Casini G. (2020). Oxidative Stress Induces a VEGF Autocrine Loop in the Retina: Relevance for Diabetic Retinopathy. Cells.

[72] Gray S.P., Jha J.C., Kennedy K., van Bommel E., Chew P., Szyndralewiez C., Touyz R.M., Schmidt H., Cooper M.E., Jandeleit-Dahm K.A.M. (2017). Combined NOX1/4 inhibition with GKT137831 in mice provides dose-dependent reno- and atheroprotection even in established micro- and macrovascular disease. Diabetologia.

[73] Pickering R.J., Rosado C.J., Sharma A., Buksh S., Tate M., de Haan J.B. (2018). Recent novel approaches to limit oxidative stress and inflammation in diabetic complications. Clin. Transl. Immunol..

[74] Darenskaya M.A., Kolesnikova L.I., Kolesnikov S.I. (2021). Oxidative Stress: Pathogenetic Role in Diabetes Mellitus and Its Complications and Therapeutic Approaches to Correction. Bull. Exp. Biol. Med..

[75] Lin M., Chen Y., Jin J., Hu Y., Zhou K.K., Zhu M., Le Y.Z., Ge J., Johnson R.S., Ma J.X. (2011). Ischaemia-induced retinal neovascularisation and diabetic retinopathy in mice with conditional knockout of hypoxia-inducible factor-1 in retinal Muller cells. Diabetologia.

[76] Jacob S., Streeper R.S., Fogt D.L., Hokama J.Y., Tritschler H.J., Dietze G.J., Henriksen E.J. (1996). The antioxidant alpha-lipoic acid enhances insulin-stimulated glucose metabolism in insulin-resistant rat skeletal muscle. Diabetes.

[77] Kaneto H., Kajimoto Y., Miyagawa J., Matsuoka T., Fujitani Y., Umayahara Y., Hanafusa T., Matsuzawa Y., Yamasaki Y., Hori M. (1999). Beneficial effects of antioxidants in diabetes: Possible protection of pancreatic beta-cells against glucose toxicity. Diabetes.

[78] Song D., Hutchings S., Pang C.C. (2005). Chronic N-acetylcysteine prevents fructose-induced insulin resistance and hypertension in rats. Eur. J. Pharmacol..

[79] Tanaka Y., Gleason C.E., Tran P.O., Harmon J.S., Robertson R.P. (1999). Prevention of glucose toxicity in HIT-T15 cells and Zucker diabetic fatty rats by antioxidants. Proc. Natl. Acad. Sci. USA.

[80] Lee H.Y., Lee J.S., Alves T., Ladiges W., Rabinovitch P.S., Jurczak M.J., Choi C.S., Shulman G.I., Samuel V.T. (2017). Mitochondrial-Targeted Catalase Protects Against High-Fat Diet-Induced Muscle Insulin Resistance by Decreasing Intramuscular Lipid Accumulation. Diabetes.

[81] Hoehn K.L., Salmon A.B., Hohnen-Behrens C., Turner N., Hoy A.J., Maghzal G.J., Stocker R., Van Remmen H., Kraegen E.W., Cooney G.J. (2009). Insulin resistance is a cellular antioxidant defense mechanism. Proc. Natl. Acad. Sci. USA.

[82] Abdel-Wahab Y.H., O’Harte F.P., Mooney M.H., Barnett C.R., Flatt P.R. (2002). Vitamin C supplementation decreases insulin glycation and improves glucose homeostasis in obese hyperglycemic (ob/ob) mice. Metab. Clin. Exp..

[83] Ma Y., Gao M., Liu D. (2016). N-acetylcysteine Protects Mice from High Fat Diet-induced Metabolic Disorders. Pharm. Res..

[84] Tanaka Y., Tran P.O., Harmon J., Robertson R.P. (2002). A role for glutathione peroxidase in protecting pancreatic beta cells against oxidative stress in a model of glucose toxicity. Proc. Natl. Acad. Sci. USA.

[85] Hotta M., Tashiro F., Ikegami H., Niwa H., Ogihara T., Yodoi J., Miyazaki J. (1998). Pancreatic beta cell-specific expression of thioredoxin, an antioxidative and antiapoptotic protein, prevents autoimmune and streptozotocin-induced diabetes. J. Exp. Med..

[86] Lortz S., Tiedge M., Nachtwey T., Karlsen A.E., Nerup J., Lenzen S. (2000). Protection of insulin-producing RINm5F cells against cytokine-mediated toxicity through overexpression of antioxidant enzymes. Diabetes.

[87] Park L., Min D., Kim H., Park J., Choi S., Park Y. (2011). The combination of metallothionein and superoxide dismutase protects pancreatic beta cells from oxidative damage. Diabetes Metab. Res. Rev..

[88] Rochette L., Vergely C., Laher I. (2014). Antioxidants and Diabetes. Systems Biology of Free Radicals and Antioxidants.

[89] Coudriet G.M., Delmastro-Greenwood M.M., Previte D.M., Marré M.L., O’Connor E.C., Novak E.A., Vincent G., Mollen K.P., Lee S., Dong H.H. (2017). Treatment with a Catalytic Superoxide Dismutase (SOD) Mimetic Improves Liver Steatosis, Insulin Sensitivity, and Inflammation in Obesity-Induced Type 2 Diabetes. Antioxidants.

[90] Brestoff J.R., Brodsky T., Sosinsky A.Z., McLoughlin R., Stansky E., Fussell L., Sheppard A., DiSanto-Rose M., Kershaw E.E., Reynolds T.H.t. (2015). Manganese [III] Tetrakis [5,10,15,20]-Benzoic Acid Porphyrin Reduces Adiposity and Improves Insulin Action in Mice with Pre-Existing Obesity. PLoS ONE.

[91] Guo J., Liu H., Zhao D., Pan C., Jin X., Hu Y., Gao X., Rao P., Liu S. (2022). Glucose-lowering effects of orally administered superoxide dismutase in type 2 diabetic model rats. npj Sci. Food.

[92] Hong Y.A., Lim J.H., Kim M.Y., Kim Y., Park H.S., Kim H.W., Choi B.S., Chang Y.S., Kim H.W., Kim T.Y. (2018). Extracellular Superoxide Dismutase Attenuates Renal Oxidative Stress Through the Activation of Adenosine Monophosphate-Activated Protein Kinase in Diabetic Nephropathy. Antioxid. Redox Signal..

[93] Moriscot C., Candel S., Sauret V., Kerr-Conte J., Richard M.J., Favrot M.C., Benhamou P.Y. (2007). MnTMPyP, a metalloporphyrin-based superoxide dismutase/catalase mimetic, protects INS-1 cells and human pancreatic islets from an in vitro oxidative challenge. Diabetes Metab..

[94] Polianskyte-Prause Z., Tolvanen T.A., Lindfors S., Kon K., Hautala L.C., Wang H., Wada T., Tsuneki H., Sasaoka T., Lehtonen S. (2022). Ebselen enhances insulin sensitivity and decreases oxidative stress by inhibiting SHIP2 and protects from inflammation in diabetic mice. Int. J. Biol. Sci..

[95] Mahadevan J., Parazzoli S., Oseid E., Hertzel A.V., Bernlohr D.A., Vallerie S.N., Liu C.Q., Lopez M., Harmon J.S., Robertson R.P. (2013). Ebselen treatment prevents islet apoptosis, maintains intranuclear Pdx-1 and MafA levels, and preserves β-cell mass and function in ZDF rats. Diabetes.

[96] Chew P., Yuen D.Y., Stefanovic N., Pete J., Coughlan M.T., Jandeleit-Dahm K.A., Thomas M.C., Rosenfeldt F., Cooper M.E., de Haan J.B. (2010). Antiatherosclerotic and renoprotective effects of ebselen in the diabetic apolipoprotein E/GPx1-double knockout mouse. Diabetes.

[97] Avignon A., Hokayem M., Bisbal C., Lambert K. (2012). Dietary antioxidants: Do they have a role to play in the ongoing fight against abnormal glucose metabolism?. Nutrition.

[98] Thakur P., Kumar A., Kumar A. (2018). Targeting oxidative stress through antioxidants in diabetes mellitus. J. Drug Target..

[99] Steinhubl S.R. (2008). Why have antioxidants failed in clinical trials?. Am. J. Cardiol..

[100] Chandil N., Pande S., Sen S.S., Gupta D. (2019). Comparison of Metformin and N Acetylcysteine on Clinical, Metabolic Parameter and Hormonal Profile in Women with Polycystic Ovarian Syndrome. J. Obstet. Gynecol. India.

[101] Fulghesu A.M., Ciampelli M., Muzj G., Belosi C., Selvaggi L., Ayala G.F., Lanzone A. (2002). N-acetyl-cysteine treatment improves insulin sensitivity in women with polycystic ovary syndrome. Fertil. Steril..

[102] Sekhar R.V. (2022). GlyNAC (Glycine and N-Acetylcysteine) Supplementation Improves Impaired Mitochondrial Fuel Oxidation and Lowers Insulin Resistance in Patients with Type 2 Diabetes: Results of a Pilot Study. Antioxidants.

[103] Yahfoufi N., Alsadi N., Jambi M., Matar C. (2018). The Immunomodulatory and Anti-Inflammatory Role of Polyphenols. Nutrients.

[104] Liu S., Lee I.M., Song Y., Van Denburgh M., Cook N.R., Manson J.E., Buring J.E. (2006). Vitamin E and risk of type 2 diabetes in the women’s health study randomized controlled trial. Diabetes.

[105] Song Y., Cook N.R., Albert C.M., Van Denburgh M., Manson J.E. (2009). Effects of vitamins C and E and beta-carotene on the risk of type 2 diabetes in women at high risk of cardiovascular disease: A randomized controlled trial. Am. J. Clin. Nutr..

[106] Czernichow S., Couthouis A., Bertrais S., Vergnaud A.C., Dauchet L., Galan P., Hercberg S. (2006). Antioxidant supplementation does not affect fasting plasma glucose in the Supplementation with Antioxidant Vitamins and Minerals (SU.VI.MAX) study in France: Association with dietary intake and plasma concentrations. Am. J. Clin. Nutr..

[107] Ashor A.W., Brown R., Keenan P.D., Willis N.D., Siervo M., Mathers J.C. (2019). Limited evidence for a beneficial effect of vitamin C supplementation on biomarkers of cardiovascular diseases: An umbrella review of systematic reviews and meta-analyses. Nutr. Res..

[108] Ashor A.W., Werner A.D., Lara J., Willis N.D., Mathers J.C., Siervo M. (2017). Effects of vitamin C supplementation on glycaemic control: A systematic review and meta-analysis of randomised controlled trials. Eur. J. Clin. Nutr..

[109] Kandhare A.D., Mukherjee A., Bodhankar S.L. (2017). Antioxidant for treatment of diabetic nephropathy: A systematic review and meta-analysis. Chem. Biol. Interact..

[110] Xu R., Zhang S., Tao A., Chen G., Zhang M. (2014). Influence of vitamin E supplementation on glycaemic control: A meta-analysis of randomised controlled trials. PLoS ONE.

[111] Suksomboon N., Poolsup N., Sinprasert S. (2011). Effects of vitamin E supplementation on glycaemic control in type 2 diabetes: Systematic review of randomized controlled trials. J. Clin. Pharm. Ther..

[112] Mason S.A., Keske M.A., Wadley G.D. (2021). Effects of Vitamin C Supplementation on Glycemic Control and Cardiovascular Risk Factors in People With Type 2 Diabetes: A GRADE-Assessed Systematic Review and Meta-analysis of Randomized Controlled Trials. Diabetes Care.

[113] Montero D., Walther G., Stehouwer C.D., Houben A.J., Beckman J.A., Vinet A. (2014). Effect of antioxidant vitamin supplementation on endothelial function in type 2 diabetes mellitus: A systematic review and meta-analysis of randomized controlled trials. Obes. Rev..

[114] Balbi M.E., Tonin F.S., Mendes A.M., Borba H.H., Wiens A., Fernandez-Llimos F., Pontarolo R. (2018). Antioxidant effects of vitamins in type 2 diabetes: A meta-analysis of randomized controlled trials. Diabetol. Metab. Syndr..

[115] Khodaeian M., Tabatabaei-Malazy O., Qorbani M., Farzadfar F., Amini P., Larijani B. (2015). Effect of vitamins C and E on insulin resistance in diabetes: A meta-analysis study. Eur. J. Clin. Investig..

[116] Tabatabaei-Malazy O., Nikfar S., Larijani B., Abdollahi M. (2014). Influence of ascorbic acid supplementation on type 2 diabetes mellitus in observational and randomized controlled trials; a systematic review with meta-analysis. J. Pharm. Pharm. Sci..

[117] Stranges S., Marshall J.R., Natarajan R., Donahue R.P., Trevisan M., Combs G.F., Cappuccio F.P., Ceriello A., Reid M.E. (2007). Effects of long-term selenium supplementation on the incidence of type 2 diabetes: A randomized trial. Ann. Intern. Med..

[118] Beckman J.A., Goldfine A.B., Leopold J.A., Creager M.A. (2016). Ebselen does not improve oxidative stress and vascular function in patients with diabetes: A randomized, crossover trial. Am. J. Physiol. Heart Circ. Physiol..

[119] Samuni Y., Goldstein S., Dean O.M., Berk M. (2013). The chemistry and biological activities of N-acetylcysteine. Biochim. Biophys. Acta.

[120] Dodd S., Dean O., Copolov D.L., Malhi G.S., Berk M. (2008). N-acetylcysteine for antioxidant therapy: Pharmacology and clinical utility. Expert. Opin. Biol. Ther..

[121] Dludla P.V., Dias S.C., Obonye N., Johnson R., Louw J., Nkambule B.B. (2018). A Systematic Review on the Protective Effect of N-Acetyl Cysteine Against Diabetes-Associated Cardiovascular Complications. Am. J. Cardiovasc. Drugs.

[122] Martina V., Masha A., Gigliardi V.R., Brocato L., Manzato E., Berchio A., Massarenti P., Settanni F., Della Casa L., Bergamini S. (2008). Long-term N-acetylcysteine and L-arginine administration reduces endothelial activation and systolic blood pressure in hypertensive patients with type 2 diabetes. Diabetes Care.

[123] Treweeke A.T., Winterburn T.J., Mackenzie I., Barrett F., Barr C., Rushworth G.F., Dransfield I., MacRury S.M., Megson I.L. (2012). N-Acetylcysteine inhibits platelet-monocyte conjugation in patients with type 2 diabetes with depleted intraplatelet glutathione: A randomised controlled trial. Diabetologia.

[124] Falach-Malik A., Rozenfeld H., Chetboun M., Rozenberg K., Elyasiyan U., Sampson S.R., Rosenzweig T. (2016). N-Acetyl-L-Cysteine inhibits the development of glucose intolerance and hepatic steatosis in diabetes-prone mice. Am. J. Transl. Res..

[125] Nair A.B., Jacob S. (2016). A simple practice guide for dose conversion between animals and human. J. Basic Clin. Pharm..

[126] Koechlin C., Couillard A., Simar D., Cristol J.P., Bellet H., Hayot M., Prefaut C. (2004). Does oxidative stress alter quadriceps endurance in chronic obstructive pulmonary disease?. Am. J. Respir. Crit. Care Med..

[127] Ferreira L.F., Campbell K.S., Reid M.B. (2011). N-acetylcysteine in handgrip exercise: Plasma thiols and adverse reactions. Int. J. Sport. Nutr. Exerc. Metab..

[128] Zheng J., Yuan X., Zhang C., Jia P., Jiao S., Zhao X., Yin H., Du Y., Liu H. (2019). N-Acetylcysteine alleviates gut dysbiosis and glucose metabolic disorder in high-fat diet-fed mice. J. Diabetes.

[129] El Sharkwy I.A., Abd El Aziz W.M. (2019). Randomized controlled trial of N-acetylcysteine versus l-carnitine among women with clomiphene-citrate-resistant polycystic ovary syndrome. Int. J. Gynaecol. Obstet..

[130] Panahi Y., Ostadmohammadi V., Raygan F., Sharif M.R., Sahebkar A. (2022). The effects of N-acetylcysteine administration on metabolic status and serum adiponectin levels in patients with metabolic syndrome: A randomized, double-blind, placebo-controlled trial. J. Funct. Foods.

[131] Sies H., Jones D.P. (2020). Reactive oxygen species (ROS) as pleiotropic physiological signalling agents. Nat. Rev. Mol. Cell Biol..

[132] Hancock J.T. (2010). Cell Signalling.

[133] Xiao H., Jedrychowski M.P., Schweppe D.K., Huttlin E.L., Yu Q., Heppner D.E., Li J., Long J., Mills E.L., Szpyt J. (2020). A Quantitative Tissue-Specific Landscape of Protein Redox Regulation during Aging. Cell.

[134] Rudyk O., Eaton P. (2014). Biochemical methods for monitoring protein thiol redox states in biological systems. Redox Biol..

[135] Forman H.J., Torres M., Fukuto J. (2002). Redox signaling. Mol. Cell Biochem..

[136] Brandes N., Schmitt S., Jakob U. (2009). Thiol-based redox switches in eukaryotic proteins. Antioxid. Redox Signal..

[137] Ghezzi P. (2013). Protein glutathionylation in health and disease. Biochim. Biophys. Acta.

[138] Musaogullari A., Chai Y.C. (2020). Redox Regulation by Protein S-Glutathionylation: From Molecular Mechanisms to Implications in Health and Disease. Int. J. Mol. Sci..

[139] Lermant A., Murdoch C.E. (2019). Cysteine Glutathionylation Acts as a Redox Switch in Endothelial Cells. Antioxidants.

[140] Lennicke C., Cocheme H.M. (2021). Redox regulation of the insulin signalling pathway. Redox Biol..

[141] Goldstein B.J., Mahadev K., Wu X., Zhu L., Motoshima H. (2005). Role of insulin-induced reactive oxygen species in the insulin signaling pathway. Antioxid. Redox Signal..

[142] Goldstein B.J. (2001). Protein-tyrosine phosphatase 1B (PTP1B): A novel therapeutic target for type 2 diabetes mellitus, obesity and related states of insulin resistance. Curr. Drug Targets Immune Endocr. Metabol. Disord..

[143] Argaev-Frenkel L., Rosenzweig T. (2022). Complexity of NAC Action as an Antidiabetic Agent: Opposing Effects of Oxidative and Reductive Stress on Insulin Secretion and Insulin Signaling. Int. J. Mol. Sci..

[144] Higaki Y., Mikami T., Fujii N., Hirshman M.F., Koyama K., Seino T., Tanaka K., Goodyear L.J. (2008). Oxidative stress stimulates skeletal muscle glucose uptake through a phosphatidylinositol 3-kinase-dependent pathway. Am. J. Physiol. Endocrinol. Metab..

[145] Czech M.P., Lawrence J.C., Lynn W.S. (1974). Evidence for the involvement of sulfhydryl oxidation in the regulation of fat cell hexose transport by insulin. Proc. Natl. Acad. Sci. USA.

[146] Krieger-Brauer H.I., Kather H. (1995). The stimulus-sensitive H_2_O_2_-generating system present in human fat-cell plasma membranes is multireceptor-linked and under antagonistic control by hormones and cytokines. Biochem. J..

[147] Goldstein B.J., Mahadev K., Wu X. (2005). Redox paradox: Insulin action is facilitated by insulin-stimulated reactive oxygen species with multiple potential signaling targets. Diabetes.

[148] Mahadev K., Zilbering A., Zhu L., Goldstein B.J. (2001). Insulin-stimulated hydrogen peroxide reversibly inhibits protein-tyrosine phosphatase 1b in vivo and enhances the early insulin action cascade. J. Biol. Chem..

[149] Schmitt T.L., Hotz-Wagenblatt A., Klein H., Droge W. (2005). Interdependent regulation of insulin receptor kinase activity by ADP and hydrogen peroxide. J. Biol. Chem..

[150] Salmeen A., Andersen J.N., Myers M.P., Meng T.C., Hinks J.A., Tonks N.K., Barford D. (2003). Redox regulation of protein tyrosine phosphatase 1B involves a sulphenyl-amide intermediate. Nature.

[151] Li P., Liu S., Lu M., Bandyopadhyay G., Oh D., Imamura T., Johnson A.M.F., Sears D., Shen Z., Cui B. (2016). Hematopoietic-Derived Galectin-3 Causes Cellular and Systemic Insulin Resistance. Cell.

[152] Maurya M., Jaiswal A., Gupta S., Ali W., Gaikwad A.N., Dikshit M., Barthwal M.K. (2022). Galectin-3 S-glutathionylation regulates its effect on adipocyte insulin signaling. Biochim. Biophys. Acta Mol. Cell Res..

[153] Chiarugi P., Cirri P. (2003). Redox regulation of protein tyrosine phosphatases during receptor tyrosine kinase signal transduction. Trends Biochem. Sci..

[154] Ahmad F., Nidadavolu P., Durgadoss L., Ravindranath V. (2014). Critical cysteines in Akt1 regulate its activity and proteasomal degradation: Implications for neurodegenerative diseases. Free Radic. Biol. Med..

[155] Xiong Y., Uys J.D., Tew K.D., Townsend D.M. (2011). S-glutathionylation: From molecular mechanisms to health outcomes. Antioxid. Redox Signal..

[156] Guy G.R., Cairns J., Ng S.B., Tan Y.H. (1993). Inactivation of a redox-sensitive protein phosphatase during the early events of tumor necrosis factor/interleukin-1 signal transduction. J. Biol. Chem..

[157] Su Z., Burchfield J.G., Yang P., Humphrey S.J., Yang G., Francis D., Yasmin S., Shin S.Y., Norris D.M., Kearney A.L. (2019). Global redox proteome and phosphoproteome analysis reveals redox switch in Akt. Nat. Commun..

[158] Flamment M., Hajduch E., Ferré P., Foufelle F. (2012). New insights into ER stress-induced insulin resistance. Trends Endocrinol. Metab..

[159] Narasimhan K.K.S., Devarajan A., Karan G., Sundaram S., Wang Q., van Groen T., Monte F.d., Rajasekaran N.S. (2020). Reductive stress promotes protein aggregation and impairs neurogenesis. Redox Biol..

[160] Loh K., Deng H., Fukushima A., Cai X., Boivin B., Galic S., Bruce C., Shields B.J., Skiba B., Ooms L.M. (2009). Reactive oxygen species enhance insulin sensitivity. Cell Metab..

[161] Merry T.L., Tran M., Dodd G.T., Mangiafico S.P., Wiede F., Kaur S., McLean C.L., Andrikopoulos S., Tiganis T. (2016). Hepatocyte glutathione peroxidase-1 deficiency improves hepatic glucose metabolism and decreases steatohepatitis in mice. Diabetologia.

[162] McClung J.P., Roneker C.A., Mu W., Lisk D.J., Langlais P., Liu F., Lei X.G. (2004). Development of insulin resistance and obesity in mice overexpressing cellular glutathione peroxidase. Proc. Natl. Acad. Sci. USA.

[163] Misu H., Takamura T., Takayama H., Hayashi H., Matsuzawa-Nagata N., Kurita S., Ishikura K., Ando H., Takeshita Y., Ota T. (2010). A Liver-Derived Secretory Protein, Selenoprotein P, Causes Insulin Resistance. Cell Metab..

[164] Takamura T. (2020). Hepatokine Selenoprotein P-Mediated Reductive Stress Causes Resistance to Intracellular Signal Transduction. Antioxid. Redox Signal..

[165] Kobayashi H., Matsuda M., Fukuhara A., Komuro R., Shimomura I. (2009). Dysregulated glutathione metabolism links to impaired insulin action in adipocytes. Am. J. Physiol. Endocrinol. Metab..

[166] Aldini G., Altomare A., Baron G., Vistoli G., Carini M., Borsani L., Sergio F. (2018). N-Acetylcysteine as an antioxidant and disulphide breaking agent: The reasons why. Free Radic. Res..

[167] Picklo M.J., Idso J.P., Jackson M.I. (2013). S-Glutathionylation of hepatic and visceral adipose proteins decreases in obese rats. Obes. Silver Spring.

[168] Ali M.A., Eid R., Hanafi M.Y. (2018). Vitamin C and E chronic supplementation differentially affect hepatic insulin signaling in rats. Life Sci..

[169] Xirouchaki C.E., Jia Y., McGrath M.J., Greatorex S., Tran M., Merry T.L., Hong D., Eramo M.J., Broome S.C., Woodhead J.S.T. (2021). Skeletal muscle NOX4 is required for adaptive responses that prevent insulin resistance. Sci. Adv..

[170] Ristow M., Zarse K., Oberbach A., Klöting N., Birringer M., Kiehntopf M., Stumvoll M., Kahn C.R., Blüher M. (2009). Antioxidants prevent health-promoting effects of physical exercise in humans. Proc. Natl. Acad. Sci. USA.

[171] Morrison D., Hughes J., Della Gatta P.A., Mason S., Lamon S., Russell A.P., Wadley G.D. (2015). Vitamin C and E supplementation prevents some of the cellular adaptations to endurance-training in humans. Free Radic. Biol. Med..

[172] Petersen A.C., McKenna M.J., Medved I., Murphy K.T., Brown M.J., Della Gatta P., Cameron-Smith D. (2012). Infusion with the antioxidant N-acetylcysteine attenuates early adaptive responses to exercise in human skeletal muscle. Acta Physiol..

[173] Wang X.L., Yang T.B., Wei J., Lei G.H., Zeng C. (2016). Association between serum selenium level and type 2 diabetes mellitus: A non-linear dose-response meta-analysis of observational studies. Nutr. J..

[174] Kohler L.N., Florea A., Kelley C.P., Chow S., Hsu P., Batai K., Saboda K., Lance P., Jacobs E.T. (2018). Higher Plasma Selenium Concentrations Are Associated with Increased Odds of Prevalent Type 2 Diabetes. J. Nutr..

[175] Vinceti M., Filippini T., Rothman K.J. (2018). Selenium exposure and the risk of type 2 diabetes: A systematic review and meta-analysis. Eur. J. Epidemiol..

[176] Mukai E., Fujimoto S., Inagaki N. (2022). Role of Reactive Oxygen Species in Glucose Metabolism Disorder in Diabetic Pancreatic beta-Cells. Biomolecules.

[177] Leloup C., Tourrel-Cuzin C., Magnan C., Karaca M., Castel J., Carneiro L., Colombani A.L., Ktorza A., Casteilla L., Penicaud L. (2009). Mitochondrial reactive oxygen species are obligatory signals for glucose-induced insulin secretion. Diabetes.

[178] Rebelato E., Abdulkader F., Curi R., Carpinelli A.R. (2010). Low doses of hydrogen peroxide impair glucose-stimulated insulin secretion via inhibition of glucose metabolism and intracellular calcium oscillations. Metab. Clin. Exp..

[179] Pi J., Bai Y., Zhang Q., Wong V., Floering L.M., Daniel K., Reece J.M., Deeney J.T., Andersen M.E., Corkey B.E. (2007). Reactive oxygen species as a signal in glucose-stimulated insulin secretion. Diabetes.

[180] Oliveira H.R., Verlengia R., Carvalho C.R., Britto L.R., Curi R., Carpinelli A.R. (2003). Pancreatic beta-cells express phagocyte-like NAD(P)H oxidase. Diabetes.

[181] Morgan D., Rebelato E., Abdulkader F., Graciano M.F., Oliveira-Emilio H.R., Hirata A.E., Rocha M.S., Bordin S., Curi R., Carpinelli A.R. (2009). Association of NAD(P)H oxidase with glucose-induced insulin secretion by pancreatic beta-cells. Endocrinology.

[182] Krippeit-Drews P., Lang F., Haussinger D., Drews G. (1994). H2O2 induced hyperpolarization of pancreatic B-cells. Pflug. Arch..

[183] Martens G.A., Cai Y., Hinke S., Stange G., Van de Casteele M., Pipeleers D. (2005). Glucose suppresses superoxide generation in metabolically responsive pancreatic beta cells. J. Biol. Chem..

[184] Argaev Frenkel L., Rozenfeld H., Rozenberg K., Sampson S.R., Rosenzweig T. (2019). N-Acetyl-l-Cysteine Supplement in Early Life or Adulthood Reduces Progression of Diabetes in Nonobese Diabetic Mice. Curr. Dev. Nutr..

[185] Chatham J.C., Gilbert H.F., Radda G.K. (1989). The metabolic consequences of hydroperoxide perfusion on the isolated rat heart. Eur. J. Biochem..

[186] Bulteau A.L., Ikeda-Saito M., Szweda L.I. (2003). Redox-dependent modulation of aconitase activity in intact mitochondria. Biochemistry.

[187] Ammon H.P., Grimm A., Lutz S., Wagner-Teschner D., Handel M., Hagenloh I. (1980). Islet glutathione and insulin release. Diabetes.

[188] Oliveira H.R., Curi R., Carpinelli A.R. (1999). Glucose induces an acute increase of superoxide dismutase activity in incubated rat pancreatic islets. Am. J. Physiol..

[189] Rebelato E., Abdulkader F., Curi R., Carpinelli A.R. (2011). Control of the intracellular redox state by glucose participates in the insulin secretion mechanism. PLoS ONE.

[190] Roma L.P., Jonas J.C. (2020). Nutrient Metabolism, Subcellular Redox State, and Oxidative Stress in Pancreatic Islets and beta-Cells. J. Mol. Biol..

[191] Munhoz A.C., Riva P., Simoes D., Curi R., Carpinelli A.R. (2016). Control of Insulin Secretion by Production of Reactive Oxygen Species: Study Performed in Pancreatic Islets from Fed and 48-Hour Fasted Wistar Rats. PLoS ONE.

[192] Weaver J.R., Grzesik W., Taylor-Fishwick D.A. (2015). Inhibition of NADPH oxidase-1 preserves beta cell function. Diabetologia.

[193] Li N., Li B., Brun T., Deffert-Delbouille C., Mahiout Z., Daali Y., Ma X.J., Krause K.H., Maechler P. (2012). NADPH oxidase NOX2 defines a new antagonistic role for reactive oxygen species and cAMP/PKA in the regulation of insulin secretion. Diabetes.

[194] Chia L.L., Jantan I., Chua K.H. (2017). Tocotrienols Stimulate Insulin Secretion of Rat Pancreatic Isolated Islets in a Dynamic Culture. Curr. Pharm. Biotechnol..

[195] Ihara Y., Yamada Y., Toyokuni S., Miyawaki K., Ban N., Adachi T., Kuroe A., Iwakura T., Kubota A., Hiai H. (2000). Antioxidant alpha-tocopherol ameliorates glycemic control of GK rats, a model of type 2 diabetes. FEBS Lett..

[196] Jin H.M., Zhou D.C., Gu H.F., Qiao Q.Y., Fu S.K., Liu X.L., Pan Y. (2013). Antioxidant N-Acetylcysteine Protects Pancreatic β-Cells Against Aldosterone-Induced Oxidative Stress and Apoptosis in Female db/db Mice and Insulin-Producing MIN6 Cells. Endocrinology.

[197] Schuurman M., Wallace M., Sahi G., Barillaro M., Zhang S., Rahman M., Sawyez C., Borradaile N., Wang R. (2022). N-acetyl-L-cysteine treatment reduces beta-cell oxidative stress and pancreatic stellate cell activity in a high fat diet-induced diabetic mouse model. Front. Endocrinol..

[198] Roma L.P., Oliveira C.A.M., Carneiro E.M., Albuquerque G.G., Boschero A.C., Souza K.L.A. (2011). N-acetylcysteine protects pancreatic islet against glucocorticoid toxicity. Redox Rep..

[199] Sánchez-Rodríguez M.A., Mendoza-Núñez V.M. (2019). Oxidative Stress Indexes for Diagnosis of Health or Disease in Humans. Oxidative Med. Cell Longev..

[200] Frijhoff J., Winyard P.G., Zarkovic N., Davies S.S., Stocker R., Cheng D., Knight A.R., Taylor E.L., Oettrich J., Ruskovska T. (2015). Clinical Relevance of Biomarkers of Oxidative Stress. Antioxid. Redox Signal..

